# Applications of reconstituted inflammasomes in a cell-free system to drug discovery and elucidation of the pathogenesis of autoinflammatory diseases

**DOI:** 10.1186/s41232-017-0040-y

**Published:** 2017-05-03

**Authors:** Naoe Kaneko, Tomoyuki Iwasaki, Yuki Ito, Hiroyuki Takeda, Tatsuya Sawasaki, Shinnosuke Morikawa, Naoko Nakano, Mie Kurata, Junya Masumoto

**Affiliations:** 1 Department of Pathology, Ehime University Graduate School of Medicine and Proteo-Science Center, Shitsukawa 454, Toon, 791-0295 Ehime Japan; 2 Divison of Proteo-Drug-Discovery Sciences, Ehime University Proteo-Science Center, Bunkyocho 3, Matsuyama, 790-8577 Ehime Japan; 3 Division of Cell-free Sciences, Ehime University Proteo-Science Center, Bunkyocho 3, Matsuyama, 790-8577 Ehime Japan

**Keywords:** Cell-free, Interleukin-1β, Inflammasome

## Abstract

The inflammasome, typically consisting of a Nod-like receptor, apoptosis-associated speck-like protein, and pro-caspase-1, has recently been identified as a huge intracellular complex, which plays a crucial role in interleukin-1 maturation or specific physiological functions. Two Nod-like receptors, such as nucleotide-binding oligomerization domains-containing protein (Nod)1 and Nod2, interact with the receptor-interacting protein serine-threonine kinase (RIPK)2 accompanied by Iκ-B kinase (IKK) complexes to construct the nodosome, leading to nuclear factor (NF)-κB activation. The aberrant activation of inflammasomes or nodosomes causes autoinflammatory diseases. Therefore, inflammasomes may be attractive targets to treat autoinflammatory diseases. Our aim is to develop reconstituted inflammasomes in a cell-free system to discover specific molecular-target drugs and elucidate the molecular pathogenesis of autoinflammatory diseases. In this review, we describe reconstituted inflammasomes in a cell-free system.

## Background

Inflammasomes have recently been identified as expanding intracellular complexes that play important roles not only in innate immunity but also in maintaining specific physiological functions [1–3]. The aberrant activation of inflammasomes is thought to be linked to various diseases, including inflammatory diseases, degenerative diseases, and tumors [4, 5]. Therefore, inflammasomes may be attractive targets to treat these diseases. Autoinflammatory diseases are known to be caused by genetic mutations of inflammasome components [6–9]. Thus, we aim to develop reconstituted inflammasomes in a cell-free system in order to identify specific molecular-target drugs and elucidate the molecular pathogenesis of autoinflammatory diseases. In this review, we briefly describe the functions of several inflammasomes and related diseases, and reconstituted inflammasomes in a cell-free system.

## General functions of inflammasomes and related diseases

The inflammasomes have been known as interleukin (IL)-1β processing platforms [10, 11]. There are several well-characterized inflammasomes: NACHT, LRR (NLR), and PYD domain-containing protein (NLRP)1 inflammasome [11], NLRP3 inflammasome [12], absent in melanoma (AIM)2 inflammasome [13–16], NLR and CARD domain-containing protein (NLRC)4 inflammasome [17], and pyrin inflammasome [18]. The inflammasome typically consists of an intracellular pathogen pattern-recognition receptor, an adaptor protein apoptosis-associated speck-like protein containing a caspase recruitment domain (ASC), and pro-caspase-1.

The NLRP1 inflammasome was the first described inflammasome to be described [11]. It has been reported to be activated by muramyl dipeptide (MDP), anthrax lethal toxins, and related to neuronal diseases [19].

The NLRP3 inflammasome is a prototype inflammasome, activated by various pathogen-associated molecular pattern molecules (PAMPs) and damage-associated molecular pattern molecules (DAMPs) [20]. NLRP3-activated PAMPs have been reported to include bacterium-derived pore-forming toxins, lethal toxins, flagellin/rod proteins, MDP, RNA, DNA, virus-derived RNA, M2 protein, fungus-derived β-glucans, hypha mannan, zymosan, and protozoon-derived hemozoin [21]. NLRP3-activated DAMPs include self-derived ATP, cholesterol crystals, monosodium urate (MSU) crystals, calcium pyrophosphate dihydrate (CPPD) crystals, glucose, β-amyloid, hyaluronic acid, and environment-derived alum, asbestos, silica, alloy particles, UV radiation, and skin irritants [21].

A single amino acid mutation in NLRP3 results in enhanced inflammasome activation, termed cryopyrin-associated periodic syndrome (CAPS), including familial cold autoinflammatory syndrome (FCAS), Muckle–Wells syndrome (MWS), and neonatal-onset multisystem inflammatory disease (NOMID)/chronic infantile neurologic, cutaneous, and arthritis (CINCA) syndrome, which leads to greater IL-1β secretion without DAMPs or PAMPs [22–27].

The AIM2 inflammasome consists of AIM2, ASC, and pro-caspase-1. AIM2 was originally identified as an interferon-gamma inducible gene product consisting of an N-terminal pyrin domain (PYD) and C-terminal hematopoietic interferon-inducible nuclear proteins with a 200-amino acid repeat (HIN-200) domain. AIM2 is differentially expressed following the suppression of the tumorigenic phenotype in a malignant melanoma cell line [28], and it subsequently acts as a sensor for cytoplasmic DNA, which forms an inflammasome with the ligand and ASC to activate caspase-1 [13–16]. The inappropriate recognition of cytoplasmic self-DNA by AIM2 contributes to the development of psoriasis, dermatitis, arthritis, and other autoimmune and inflammatory diseases [29].

The NLRC4 inflammasome consists of NLRC4, ASC, and pro-caspase-1. Since the protein-binding motif of NLRC4 is CARD instead of the PYD, NLRC4 interacts with ASC as well as pro-caspase-1 through their CARD. NLRC4 constitutes an inflammasome, which is required for the recognition of bacterial flagellin [30, 31]. Several mutations in the nucleotide-binding domain of NLRC4 cause autoinflammatory diseases, early-onset recurrent fever flares, and macrophage activation syndrome (MAS) [32–34].

Pyrin has been identified as a causative gene of the *MEFV* product of familial Mediterranean fever (FMF), an autosomal recessive inherited autoinflammatory syndrome [35]. Pyrin not only regulates several inflammasomes [36–38] but also constructs an inflammasome with ASC and pro-caspase-1, upon recognizing some pathogens [18, 39, 40]. Thus, FMF patients with some pyrin mutations are thought to show autosomal dominant inheritance [41–43].

## General functions of the nodosomes and related diseases

Nod1 and Nod2, both of which are involved in host recognition of small molecules, activate NF-κB in response to sensing the component of peptidoglycan [44–47]. NF-κB activation in Nod1 and Nod2 depends on RIPK2 and IKK machinery [48]. The core ligand structure of Nod2 is *N*-Acetyl muramyl-l-alanyl-d-isoglutamine hydrate, also known as MDP, of which the structure is common in bacteria. The ligand for Nod1 is a dipeptide designated as d-glutamyl-*meso*-diaminopimelic acid (*i*E-DAP), with the structure being derived from a subgroup of bacteria [44–47].

Functional activation by genetic mutations of Nod2 is associated with autoinflammatory diseases, Blau syndrome (BS), and early-onset sarcoidosis (EOS), which are characteristics of systemic granulomatous diseases [49]. However, genetic and functional defects of Nod2 are associated with susceptibility to Crohn’s disease, an inflammatory bowel disease. There is no known Nod1-related autoinflammatory disease, but associations between SNPs in NOD1 and several immune-related diseases, such as inflammatory bowel disease, atopic eczema, asthma, and rheumatoid arthritis have been reported [50–53].

## Wheat germ cell-free protein synthesis for inflammasomes

To construct reconstituted inflammasomes in a cell-free system, we employed the wheat germ cell-free protein synthesis system rather than *Escherichia coli* expression system [54]. When we identified ASC a central adaptor protein of inflammasomes, ASC was discovered in the Triton X-100-insoluble fraction of promyelocytic leukemia cell line HL-60 cells [55], and it was difficult to synthesize recombinant NLRP3 protein using *E. coli* expression due to its solubility. On the other hand, the wheat germ cell-free protein synthesis has numerous advantages, such as low cost, ease of availability in large amounts, low endogenous incorporation, and the capacity to synthesize high-molecular-weight proteins [54]. In addition, it is suitable for the expression of eukaryotic proteins because it is eukaryotic system [55].

## Reconstituted AIM2 inflammasome in a cell-free system

First, we describe an AIM2 inflammasome in a cell-free system as a prototype [56] because the AIM2 inflammasome has been well-characterized, and its ligand was reported to be present in poly-deoxyadenylic-deoxythymidylic acid, poly(dA:dT). The direct interaction between AIM2 and poly(dA:dT) was elucidated using the amplified luminescent proximity homogeneous assay (Alpha) [14]. In addition, activation of the AIM2 inflammasome has been reported to be related to various diseases [57–62], and it is thought to be an attractive drug target for diseases.

Our reconstituted AIM2 inflammasome basically consists of AIM2 and ASC, and it is considered sufficient for drug and ligand discovery as it assembles without pro-caspase-1 or any other components [56].

To synthesize the AIM2 inflammasome, PCR products for AIM2 and ASC were inserted into a Gateway™ pDONR^TM^221 Vector (pDONR221) (Life Technologies, Carlsbad, CA, USA) using Gateway™ BP Clonase™ II Enzyme mix (Life Technologies, Carlsbad, CA, USA) to generate entry clones. The AIM2 entry clone pDONR221-AIM2 was inserted into pEU-E01-GW-bls-STOP for cell-free protein expression. The ASC entry clones pDONR221-ASC and pDONR221-ASC-PYD were inserted into pEU-E01-FLAG-GW-STOP using the Gateway™ LR Clonase™ II Enzyme mix (Life Technologies, Carlsbad, CA, USA). The constructed plasmids were used to synthesize specific proteins with the WEPRO1240 Expression Kit (Cell-Free, Inc., Matsuyama, Japan) [56].

**Fig. 1:**
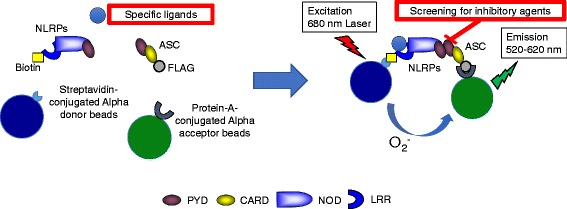
Schematic representation of reconstituted inflammasomes. Once specific ligands are recognized by NLRPs, inflammasomes are constituted. Then, chemical energy of reactive oxygen from donor beads is transferred to acceptor beads and a signal is detected

In our AIM2 inflammasome, proximity between AIM2 and ASC is detected by the Alpha using the combination of protein-A-conjugated Alpha acceptor beads for FLAG-tagged proteins and streptavidin-conjugated Alpha donor beads for biotinylated proteins (Fig. 1).

The AIM2 inflammasome in a cell-free system assembles with its previously reported ligand poly(dA:dT), and the interaction between AIM2 and ASC was disrupted by anti-human ASC mAb, and previously reported inhibitors CRID3 and glycyrrhizin. Thus, our reconstituted AIM2 inflammasome in a cell-free system is useful for investigating novel ligands and drug discovery [56].

## Reconstituted NLRP3 inflammasome in a cell-free system

When AIM2 is replaced by NLRP3, we can easily develop the NLRP3 inflammasome in a cell-free system. There are so many mutations in NLRP3 that causes of autoinflammatory diseases including CAPS, and NLRP3 involve various inflammasomopathies. Thus, the reconstituted NLRP3 inflammasome in a cell-free system will be a useful tool for investigating inflammasomopathies and drug discovery. In this context, we are going to develop reconstituted NLRP3 inflammasome in a cell-free system.

## Reconstituted Nod2 nodosome in a cell-free system

The autoinflammatory disease Blau syndrome (BS)/early-onset sarcoidosis (EOS) is caused by a point mutation of Nod2 [49]. Therefore, the Nod2 nodosome may be an attractive drug target for the treatment of BS/EOS. We aimed to develop a reconstituted protein–protein interaction assay system between wild-type Nod2 and the BS/EOS-associated mutants of Nod2 and RIPK2 in a cell-free system, called the reconstituted Nod2 nodosome in a cell-free system [63].

The plasmids vector pDONR221-Nod2 and BS/EOS-associated mutants, pDONR221-Nod2-R334W and pDONR221-Nod2-N670K, were constructed. pDONR221-RIPK2 and pDONR221-RIPK2-CARD were also constructed. Then, the proteins Nod2-WT-Btn, Nod2-R334W-Btn, Nod2-N670K-Btn, FLAG-RIPK2 and FLAG-RIPK2-CARD were synthesized using the wheat germ cell-free system in the same way as AIM2.

In our Nod2 nodosome, proximity between Nod2 and RIPK2 is basically detected by Alpha using the combination of protein-A-conjugated Alpha acceptor beads for FLAG-tagged proteins and streptavidin-conjugated Alpha donor beads for biotinylated proteins. The Nod2 nodosome in a cell-free system assembles with its previously reported ligand MDP. The Nod2 nodosomes with BS/EOS-associated mutations Nod2-R334W and Nod2-N670K were more sensitive to MDP than Nod2-WT. Therefore, we think that our Nod2 nodosome in a cell-free system can be a useful tool for investigating the pathogenesis of BS/EOS and drug discovery [63].

## How does the reconstituted inflammasome in a cell-free system work?

**Fig. 2:**
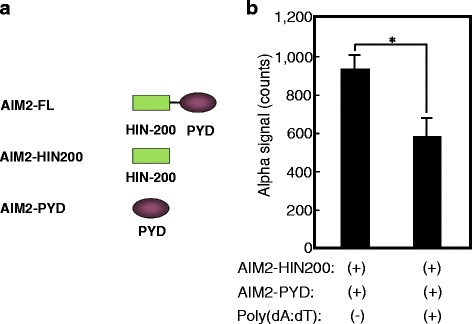
poly(dA:dT) reduces the amplified luminescent proximity signal between the PYD domain of AIM2 and the HIN-200 domain of AIM2. A schematic representation of full-length AIM2 (AIM2-FL) and its truncated proteins, the HIN200 domain of AIM2 (AIM2-HIN200) and PYD domain of AIM2 (AIM2-PYD). We synthesized two truncated forms of AIM2, AIM2-PYD-FLAG and AIM-HIN-200-Biotin, using a wheat germ cell-free synthesis system (**a**). Synthetic protein–protein interactions were detected by the amplified luminescent proximity homogeneous assay (Alpha). A total of 100 ng of each protein indicated was incubated with 5 μg/mL anti-FLAG mAb M2, 16.67 μg/mL protein-A-conjugated Alpha acceptor beads (PerkinElmer, Waltham, MA, USA), and 16.67 μg/mL streptavidin-conjugated Alpha donor beads (PerkinElmer, Waltham, MA, USA) for 24 h with or without 5 mg/mL poly(dA:dT) (Invivogen, San Diego, CA, USA). Responses (counts) were measured using EnSpire™ Multimode Plate Reader (PerkinElmer, Waltham, MA, USA). The results are given as means ± standard deviation from triplicate wells. *Asterisk* indicates significance (*p <* 0.01) (**b**)

**Fig. 3:**
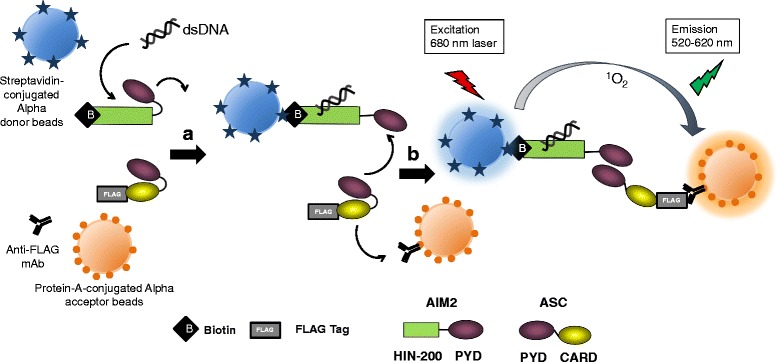
A possible mechanism of AIM2 inflammasome in a cell-free system. The PYD domain of AIM2 interacts with the HIN-200 domain of AIM2 loosely upon being incubated with no materials. Once a specific ligand poly(dA:dT) stringently interacts with the HIN-200 domain of AIM2 and streptavidin-conjugated donor beads, then the PYD domain of AIM2 apart from the HIN-200 domain of AIM2 opens to interact with the PYD domain of ASC (**a**). Since the PYD domain of ASC loosely interacts with the CARD domain of ASC, the interaction between the PYD domain of AIM2 and PYD domain of ASC is thought to open the CARD domain of ASC, which will lead to interaction with protein A-conjugated acceptor beads (**b**)

We show representative data that suggest the mechanism of how this system works (Fig. 2). We synthesized two truncated forms of AIM2, AIM2-PYD-FLAG and AIM-HIN-200-Biotin, using a wheat germ cell-free synthesis system (Fig. 2a). The amplified luminescence proximity signal between AIM2-PYD-FLAG and AIM-HIN-200-Biotin was 940.0 ± 100.5 with no materials and 626.7 ± 98.7 upon incubation with poly(dA:dT) (Invivogen, San Diego, CA, USA). The difference of signals was significant (*p =* 0.000917) using Student’s *t* test (Fig. 2b). The data suggest that the PYD domain of AIM2 interacts with the HIN-200 domain of AIM2 loosely upon being incubated with no materials. Once the specific ligand poly(dA:dT) stringently interacts with the HIN-200 domain of AIM2, then the PYD domain of AIM2 apart from the HIN-200 domain of AIM2 is open to interact with the PYD domain of ASC. Since the PYD domain of ASC has been reported to loosely interact with the CARD domain of ASC [64], the interaction between the PYD domain of AIM2 and PYD domain of ASC is thought to open the CARD domain of ASC, which will lead to interaction with protein A-conjugated acceptor beads in the cell-free system (Fig. 3), or downstream CARD domain of caspase-1 in cells.

## Conclusions

Various inflammasomes are thought to play important roles in the maintenance of the homeostasis of cells, tissues, and organs. Excess inflammasome signaling caused by genetic mutations or pathogens may contribute to known or unknown autoinflammatory diseases. Thus, inflammasomes are expected to become attractive targets to treat autoinflammatory diseases. Although our cell-free system is limited in that only an initial event of assembly between ASC or RIPK2 and an upstream protein is detected, reconstituted inflammasomes in a cell-free system will be useful tools for investigating the pathogenesis of autoinflammatory diseases and discovery of their therapeutics.
